# Prevalence and risk factors for abnormal tandem gait in patients with essential tremor syndrome: A cross-sectional study in Southwest China

**DOI:** 10.3389/fneur.2023.998205

**Published:** 2023-02-16

**Authors:** Hongyan Huang, Xianghua He, Qiuyan Shen, Dan Zhang, Yi Bao, Fang Xu, Anling Luo, Ling Liu, Xinglong Yang, Yanming Xu

**Affiliations:** ^1^Department of Neurology, West China Hospital, Sichuan University, Chengdu, Sichuan, China; ^2^Department of Neurology, Jiangbin Hospital, Nanning, Guangxi, China; ^3^Department of Geriatric Neurology, First Affiliated Hospital of Kunming Medical University, Kunming, Yunnan, China

**Keywords:** essential tremor syndrome, balance impairments, tandem gait, depression, falls, near falls

## Abstract

**Objectives:**

Patients with essential tremor (ET) syndrome have more prevalent and more serious gait and balance impairments than healthy controls. In this cross-sectional study, we explored whether balance impairments are associated with falls as well as more pronounced non-motor symptoms in patients with ET syndrome.

**Methods:**

We assessed the tandem gait (TG) test, as well as falls or near-falls that occurred over the previous year. Non-motor symptoms—including cognitive deficits, psychological and sleep disorders—were evaluated. In univariate analyses, statistical significance was corrected for multiple comparisons using the Benjamini–Hochberg method. Multiple logistic regression was utilized to evaluate the risk factors of poor TG performance in patients with ET syndrome.

**Results:**

A total of 358 patients with ET syndrome were divided into the abnormal TG (a-TG) and normal TG (n-TG) groups based on their performances in the TG test. We revealed that 47.2% of patients with ET syndrome had a-TG. The patients with a-TG were older, were more likely female, and were more likely present with cranial tremors and falls or near-falls (all adjusted *P* < 0.01). The patients with a-TG had significantly lower Mini-Mental Status Examination scores, as well as significantly higher Hamilton Depression/Anxiety Rating Scale and Pittsburgh Sleep Quality Index scores. Multiple logistic regression analysis demonstrated that female sex (OR 1.913, 95% CI: 1.180–3.103), age (OR 1.050, 95% CI: 1.032–1.068), cranial tremor scores (OR 1.299, 95% CI: 1.095–1.542), a history of falls or near-falls (OR 2.952, 95% CI: 1.558–5.594), and the presence of depressive symptoms (OR 1.679, 95% CI: 1.034–2.726) were associated with the occurrence of a-TG in patients with ET syndrome.

**Conclusion:**

TG abnormalities may be a predictor of fall risk in patients with ET syndrome and are associated with non-motor symptoms, especially depression.

## 1. Introduction

Essential tremor (ET) syndrome (including “pure” ET and ET-plus) mainly manifests as postural or kinetic tremors in the upper limbs and is the most prevalent movement disorder worldwide (1). In addition to tremor symptoms, patients with ET syndrome also present varied non-motor symptoms (e.g., cognitive deficits, mental disorders, and sleep disruptions) and “soft” signs [e.g., tandem gait (TG) impairment, rest tremor, and slight rigidity] (1).

Balance and gait impairments in ET syndrome were initially reported by Singer et al. in 1994, who revealed the poor performance of patients with ET syndrome in the TG test compared to that of controls (2). Subsequent studies—*via* bedside neurologic exams, subjective or objective scales, posturographies, and/or quantitative gait analyses—have yielded similar conclusions that balance and gait impairments are more common in patients with ET syndrome than in controls (3–8). Moreover, several factors have been associated with balance and gait impairments in ET syndrome, including age, late onset of tremor, disease duration, presence of cranial tremors, presence of intention tremor, and cognitive deficits (3–8).

In addition to cognitive deficits, more prominent depression, anxiety, and sleep problems have been widely reported in patients with ET syndrome (9). Prospective studies have suggested that depression and cognitive impairments may precede tremor symptoms and may represent the primary symptoms among patients with ET syndrome (9). A recent imaging study by Sengul et al. (10) revealed that patients with ET syndrome, depression, and anxiety exhibit structural changes in the amygdala, ventrolateral prefrontal cortex, and precuneus. Moreover, the amygdala and other limbic structures are linked *via* efferent projections to regions such as the basal ganglia, reticular formation, and vestibular nuclei, all of which are involved in postural and balance control (11). Meanwhile, higher levels of depressive symptoms related to poor performance in quantitative gait analysis have been revealed in community-dwelling elderly individuals (12). Furthermore, both subjective and objective sleep disturbances are associated with the risk of falls, fear of falling, and postural instability in elderly individuals (13, 14). However, the correlations and interactions among balance/gait impairments and mental/sleep disorders have not yet been investigated in patients with ET syndrome. We speculate that these non-motor symptoms may accompany poor balance in patients with ET syndrome.

Balance impairments may result in a higher risk of falls which may increase the burden on caregivers as well as reduce patients' quality of life. However, the burden and risk factors for balance impairments among the Chinese ET syndrome population remain unknown. In the present study, we employed the TG test (15), a sensitive and utility bedside neurologic exam for evaluating balance, to determine balance functionality in ET syndrome in Southwest China. Furthermore, we explored the incidence of balance impairments and their associations with tremor symptoms, and near-falls/falls, as well as with cognitive, anxious, depressive, and sleep symptoms.

## 2. Patients and methods

### 2.1. Participants

A total of 358 drug-naïve patients with ET syndrome who did not have dementia were enrolled in an ongoing (2016–2022) cross-sectional clinical study in the Department of Neurology at West China Hospital, which is affiliated with Sichuan University (Chengdu, China). Brain magnetic resonance imaging (MRI) and thyroid function tests were conducted in all patients. Probable or definite ET syndrome was diagnosed by the procedure of the Movement Disorder Society on Tremor (consensus criteria in 1998) and was based on examinations by two neurologists (HYH and YMX) (16). Patients were excluded if they had (a) tremor duration shorter than 3 years; (b) tremor related to other central nervous system diseases, such as Parkinson's disease, Wilson's disease, and dystonia; (c) tremor related to isolated focal tremor (i.e., isolated head tremor), enhanced physiologic tremor, psychogenic tremor, orthostatic tremor, task-specific tremor, drug intake, and alcohol withdrawal; and (d) conditions considered likely to limit gait or balance (i.e., peripheral neuropathies, severe visual impairments, and disabling osteoarthrosis). The present study was approved by the Ethics Committee of West China Hospital, Sichuan University (2020-842), and each participant signed an informed consent form.

### 2.2. Evaluation of tremors

We used relevant sections from the Fahn–Talosa–Marin Tremor Rating Scale (FTM-TRS)—which contains parts A, B, and C—to assess limb tremors and cranial tremors (17). For limb tremor scoring, we summed the postural/kinetic tremor scores of items 5 and 6 in part A and all items in part B for upper-limb-action tremor scores, whereas we summed the postural/kinetic tremor scores of items 8 and 9 in part A for lower-limb-action tremor scores. Limb rest tremor scores were calculated from the rest of the tremor part of items 5 and 6 and items 8 and 9 in part A.

We evaluated the neck, voice, and facial tremors using items 1, 3, and 4 in part A (17), respectively, and their sum represented the cranial tremor score. When scored for at least one point of a body part or a tremor type, the patients were considered to have the presence of a tremor in that corresponding body part or tremor type.

Intention tremor, evaluated by the finger-nose-finger test, was defined as tremor amplitude that increased during movements close to the target (4). The presence of intention tremor was defined as having either probable intention tremor in both arms or definite/incapacitating intention tremor in at least one arm (4).

### 2.3. Gait and falls

The TG test was implemented to evaluate the balance functionality of each patient. The TG task consists of alternating between putting one foot in front of the other and touching the toe to heel with arms at one's sides and with one's eyes being open (2–5, 15). The misstep was when the forward foot failed to land ahead of and close to the toes of the backward foot (out-of-line) (2). Each participant performed three 10-step TG trials, during which missteps in every turn were recorded. Patients were grouped as having a normal TG (n-TG) or abnormal TG (a-TG) based on the findings that two or more missteps per trial occurred and the findings had to be reproduced two times or more than that (2–5, 18).

We also recorded the history of falls or near-falls that occurred in the past year. A fall occurred neither with external perturbation nor as the result of a major intrinsic event such as stroke or syncope. A near-fall was defined as a patient's feeling that he/she is going to fall but does not actually fall down (6).

### 2.4. Non-motor symptoms

We used the Chinese version of the Mini-Mental Status Examination (CMSE) to evaluate the global cognitive function (19). Depression and anxiety were assessed using the Hamilton Depression Rating Scale with 24 items (HDRS-24) and Hamilton Anxiety Rating Scale (HARS), respectively, with an HDRS-24 score of ≥8 and a HARS score of ≥7 indicating at least mild depression and anxiety (20–22). We used the global Pittsburgh Sleep Quality Index (PSQI) and its seven components to assess sleep quality, with scores ≥6 being indicative of poorer subjective sleep quality (23).

### 2.5. Statistical analyses

Continuous variables are presented as the mean ± standard deviation (SD), as well as the median (ranges), while categorical or ordered variables are presented as counts and frequencies. As our data were not normally distributed (as determined by the Kolmogorov–Smirnov test), comparisons of continuous or ordered variables between patients with ET syndrome with n-TG and a-TG were determined using a Mann–Whitney U-test. In addition, we applied Pearson's chi-squared test or Fisher's exact test to compare categorical variables. In univariate analyses, statistical significance was corrected for multiple comparisons using the Benjamini–Hochberg method.

Spearman's correlation test was used to analyze the association between the total number of missteps during three turns and other independent variables. The association of CMSE, HDRS, HARS, and PSQI scale scores was also analyzed using Spearman's correlation test.

Due to collinearity and non-normal distribution of variables, as well as the purpose of the study being more concerned with risk factors associated with gait and balance impairments (characterized by a-TG) rather than risk factors associated with specific numbers of missteps, we used multiple binary logistic regression (likelihood-ratio forward-entry method) instead of the multiple linear regression model to explore the potential factors of a-TG in patients with ET syndrome. In our initial analysis, the model included all 358 patients with ET syndrome. In an additional analysis, we removed patients who had mild osteoarthrosis with no subjective interference on daily walking or who were currently taking medications that may directly (e.g., sedating medications) or indirectly (e.g., medications for blood pressure or diabetes mellitus) affect balance, gait, or increase the risk of falls (for medications, see Supplementary Table 1). The independent variables included the following: sex, age, age of tremor onset (< 47/≥47 years, the median age of all the patients), disease duration, educational years, CMSE scores, arm action tremor sores, cranial tremor scores, presence of intention tremor, presence of falls or near-falls, presence of anxiety symptoms (HARS score ≥7), presence of depressive symptoms (HDRS score ≥8), and presence of poorer sleep quality (PSQI score ≥6). All models generated odds ratios (ORs) and 95% confidence intervals (CIs). The tests were two-tailed, and a *p*-value of < 0.05 was considered significant. Statistical analyses were performed in SPSS 23.0 (IBM, Chicago, IL, USA).

## 3. Results

### 3.1. Demographic information

There were 358 patients (180 women, 50.3%) in the present study, with a median age of 56.0 years. All the patients had postural/kinetic tremors in their arms, and more than half of the patients (204, 57.0%) had tremors in cranial locations.

### 3.2. Clinical variables associated with balance impairment in patients with ET syndrome

A total of 169 patients (47.2%) had a-TG. The likelihood of a-TG was higher in women as well as in patients with advanced age, late age of tremor onset, fewer years of education, hypertension, and osteoarthrosis (all adjusted *P* < 0.05) (Table 1).

**Table 1:** Demographic characteristics and medical history in ET syndrome patients with normal or abnormal TG.

**Variables**	**All patients (*N =* 358)**	**Normal TG (*N =* 189)**	**Abnormal TG (*N =* 169)**	***p* ^b^**	***Adj*.*p*^b, c^**
**Demographics**					
Age	53.51 ± 16.08 (56.00)	48.11 ± 15.02 (50.00)	59.56 ± 15.08 (64.00)	< 0.001^*^	< 0.001^*^
Female gender	180 (50.3%)	76 (40.2%)	104 (61.5%)	< 0.001^*^	< 0.001^*^
Education in years	10.54 ± 4.62 (9.00)	11.27 ± 4.32 (12.00)	9.72 ± 4.82 (9.00)	0.001^*^	0.002^*^
Height	161.90 ± 7.60 (160.00)	163.69 ± 7.82 (163.00)	159.89 ± 6.82 (160.00)	< 0.001^*^	< 0.001^*^
Weight	59.87 ± 9.93 (60.00)	61.63 ± 10.48 (60.00)	57.91 ± 8.91 (58.00)	0.001^*^	0.004^*^
BMI	22.78 ± 2.97 (22.60)	22.95 ± 3.15 (22.86)	22.58 ± 2.74 (22.50)	0.387	0.444
**Medical history**					
Age of tremor onset	43.38 ± 17.28 (46.00)	38.93 ± 15.73 (41.00)	48.36 ± 17.60 (51.00)	< 0.001^*^	< 0.001^*^
Disease duration	10.15 ± 9.36 (5.00)	9.16 ± 8.63 (5.00)	11.25 ± 10.02 (7.00)	0.065	0.090
Positive family history	157 (43.9%)	79 (41.8%)	78 (46.2%)	0.687	0.458
Current smoker	72 (20.1%)	47 (24.9%)	25 (14.8%)	0.018^*^	0.031^*^
Current drinker	52 (14.5%)	29 (15.3%)	23 (13.6%)	0.642	0.667
**With comorbidity**					
Hypertension	44 (12.3%)	15 (7.9%)	29 (17.2%)	0.008^*^	0.017^*^
Heart disease	11 (3.1%)	5 (2.6%)	6 (3.6%)	0.620	0.657
Diabetes	22 (6.1%)	11 (5.8%)	11 (6.5%)	0.786	0.801
Respiratory illness	9 (2.5%)	3 (1.6%)	6 (3.6%)	0.317	0.372
Osteoarthrosis	20 (5.6%)	4 (2.1%)	16 (9.5%)	0.003^*^	0.006^*^
^a^ Currently taking medications predisposed to influence balance, gait and/or falls	51 (14.2%)	21 (11.1%)	30 (17.8%)	0.073	0.098

Values are the mean ± standard deviation and/or median or number (percentage);

^a^The medicine may directly or indirectly affect balance and gait, or increase the risk of falls (see Supplementary Table 1 for details);

^b^Comparisons of variables between the normal TG and abnormal TG groups;

^c^The p-values were adjusted using the Benjamini–Hochberg method;

p-values calculated by the Mann–Whitney U-test, chi-square test, or Fisher's exact test, ^*^Significant difference.

Tremors were more severe (higher scores of arm action tremor and cranial tremor) and more widely distributed (higher incidences of the neck, facial, and cranial tremors) in patients with ET syndrome with a-TG (all adjusted *P* < 0.05). The scores of functional disabilities related to tremors (FTM-TRS, part C) were also significantly higher in the a-TG group than in the n-TG group (adjusted *P* = 0.001) (Table 2).

**Table 2:** Motor and non-motor symptoms in ET syndrome patients with normal or abnormal TG.

**Variables**	**All patients (*N =* 358)**	**Normal TG (*N =* 189)**	**Abnormal TG (*N =* 169)**	***p* ^e^**	***AdjP* ^e, f^**
**Motor symptoms**					
Arm action tremor score	18.55 ± 9.91 (17.00)	17.52 ± 9.67 (16.00)	19.70 ± 10.07 (18.00)	0.020^*^	0.033^*^
Arm intention tremor score	0.50 (0–4)	0.50 (0–4)	0.50 (0–4)	0.269	0.323
Presence of intention tremor	126 (35.2%)	64 (33.9%)	62 (36.7%)	0.576	0.623
Rest tremor score	0.00 (0–12)	0.00 (0–4)	0.00 (0–12)	0.148	0.186
Presence of rest tremor	44 (12.3%)	19 (10.1%)	25 (14.8%)	0.173	0.212
Leg action tremor score	0.00 (0–12)	0.00 (0-8)	0.00 (0–12)	0.547	0.603
Presence of leg action tremor	149 (41.6%)	79 (41.8%)	70 (41.4%)	0.942	0.942
Functional disability score (TRS C)	6.86 ± 4.83 (6.00)	6.06 ± 4.63 (5.00)	7.75 ± 4.90 (7.00)	< 0.001^*^	0.001^*^
Neck tremor score	0.00 (0–6)	0.00 (0–4)	0.00 (0–6)	< 0.001^*^	0.002^*^
Presence of neck tremor	140 (39.1%)	60 (31.7%)	80 (47.3%)	0.003^*^	0.006^*^
Voice tremor score	0.00 (0–4)	0.00 (0-2)	0.00 (0–4)	0.037^*^	0.055
Presence of voice tremor	109 (30.4%)	50 (26.5%)	59 (34.9%)	0.083	0.109
Facial tremor score	0.00 (0–3)	0.00 (0-2)	0.00 (0–3)	< 0.001^*^	0.001^*^
Presence of facial tremor	41 (11.5%)	11 (5.8%)	30 (17.8%)	< 0.001^*^	0.001^*^
^a^ Cranial tremor grade				
0	154 (43.0%)	99 (52.4%)	55 (32.5%)	< 0.001^*^	< 0.001^*^
1	128 (35.8%)	63 (33.3%)	65 (38.5%)		
2	76 (21.2%)	27 (14.3%)	49 (29.0%)		
Cranial tremor score	1.00 (0–9)	0.00 (0–6)	1.00 (0–9)	< 0.001^*^	< 0.001^*^
Presence of cranial tremor	204 (57.0%)	90 (47.6%)	114 (67.5%)	< 0.001^*^	< 0.001^*^
**Non-motor symptoms**					
CMSE score	27.68 ± 2.46 (28.00)	28.18 ± 2.05 (29.00)	27.12 ± 2.76 (28.00)	< 0.001^*^	< 0.001^*^
HARS score	9.82 ± 6.51 (9.00)	8.68 ± 6.14 (8.00)	11.10 ± 6.70 (10.00)	0.001^*^	0.002^*^
^b^ With mild anxiety symptoms	228 (63.7%)	109 (57.7%)	119 (70.4%)	0.012^*^	0.024^*^
HDRS score	8.73 ± 6.67 (7.00)	7.41 ± 6.07 (6.00)	10.21 ± 7.01 (9.00)	< 0.001^*^	< 0.001^*^
^c^ With mild depressive symptoms	176 (49.2%)	76 (40.2%)	100 (59.2%)	< 0.001^*^	0.001^*^
PSQI				
Subjective sleep quality	1.00 (0–3)	1.00 (0–3)	1.00 (0–3)	0.025^*^	0.041^*^
Sleep latency	1.00 (0–3)	1.00 (0–3)	1.00 (0–3)	0.016^*^	0.029^*^
Sleep duration	1.00 (0–3)	1.00 (0–3)	1.00 (0–3)	0.027^*^	0.043^*^
Habitual sleep efficiency	0.00 (0–3)	0.00 (0–3)	0.00 (0–3)	0.027^*^	0.042^*^
Sleep disturbances	1.00 (0-2)	1.00 (0-2)	1.00 (0-2)	0.049^*^	0.070
Use of sleeping medication	0.00 (0–3)	0.00 (0–3)	0.00 (0–3)	0.040^*^	0.059
Daytime dysfunction	1.00 (0–3)	1.00 (0–3)	1.00 (0–3)	0.103	0.133
Global PSQI score	5.92 ± 4.22 (5.00)	5.25 ± 3.96 (4.00)	6.66 ± 4.39 (6.00)	0.002^*^	0.004^*^
^d^ With poorer sleep quality	165 (46.1%)	73 (38.6%)	92 (54.4%)	0.003^*^	0.006^*^
**Falls or near falls in the past year**					
Numbers of near falls	0.00 (0-5)	0.00 (0-2)	0.00 (0-5)	< 0.001^*^	< 0.001^*^
History of near falls	44 (12.3%)	11 (5.8%)	33 (19.5%)	< 0.001^*^	< 0.001^*^
Numbers of falls	0.00 (0-5)	0.00 (0-5)	0.00 (0–4)	0.013^*^	0.024^*^
History of falls	34 (9.5%)	11 (5.8%)	23 (13.6%)	0.012^*^	0.024^*^
Numbers of near falls or falls	0.00 (0-5)	0.00 (0-5)	0.00 (0-5)	< 0.001^*^	< 0.001^*^
History of near falls or falls	68 (19.0%)	20 (10.6%)	48 (28.4%)	< 0.001^*^	< 0.001^*^

Values are the mean ± standard deviation and/or median (range) or number (percentage);

^a^Defined as 0, without a cranial tremor; 1, cranial tremor at one position; 2, cranial tremor at multiple positions;

^b^Defined as HARS score ≥ 7;

^c^Defined as HDRS score ≥ 8;

^d^Defined as PSQI score ≥ 6;

^e^Comparisons of variables between the normal TG and abnormal TG groups;

^f^The p-values were adjusted using the Benjamini–Hochberg method;

p-values calculated by the Mann–Whitney U-test, chi-square test or Fisher's exact test, ^*^Significant difference.

Among patients who had a-TG, the proportion with a history of falls or near-falls (*n* = 48, 28.4%) was more than two times that of patients with n-TG (*n* = 20, 10.6%) (adjusted *P* < 0.001) (Table 2). Moreover, patients with a history of near-falls were more likely to experience falls than those who did not have a history of near-falls (22.7 vs. 7.6%, *P* = 0.004).

Patients with a-TG had significantly lower CMSE scores, as well as significantly higher HDRS/HARS scores and global PSQI scores (all adjusted *P* < 0.01). Among the seven components in the PSQI, patients with a-TG showed more severe sleep disorders than n-TG patients in terms of “subjective sleep quality,” “sleep latency,” “sleep duration,” and “habitual sleep efficiency” (all adjusted *P* < 0.05). Overall, the presence of anxiety symptoms (HARS score ≥7), depressive symptoms (HDRS score ≥8), or poor sleep quality (PSQI score ≥6) was significantly more frequently represented in patients with a-TG than patients with n-TG (all adjusted *P* < 0.05) (Table 2).

The correlations between the total number of missteps and clinical variables are presented in Table 3. The number of missteps had weak to moderate correlations with female sex, age, age of tremor onset, years of education, cranial tremor scores, number of near falls or falls, CMSE score, HDRS score, and PSQI score (0.2 < rs < 0.6, *P* < 0.05). In terms of non-motor symptom scales, the CMSE score showed correlations with the HDRS score (rs = −0.236), HARS score (rs = −0.204), and PSQI score (rs = −0.202); the HDRS score showed correlations with the HARS score (rs = 0.852) and PSQI score (rs = 0.664); and the HARS score showed correlation with the PSQI score (rs = 0.592) (all with *P* < 0.001).

**Table 3:** Correlation of total numbers of missteps with independent variables.

	**Total numbers of missteps**	
	**rs**	***p***
Female gender	0.228	< 0.001^*^
Age	0.491	< 0.001^*^
Age of tremor onset	0.389	< 0.001^*^
Disease duration	0.105	0.048^*^
Education in years	−0.227	< 0.001^*^
BMI	−0.022	0.674
Arm action tremor score	0.188	< 0.001^*^
Arm intention tremor score	0.142	0.007^*^
Rest tremor score	0.108	0.041^*^
Leg action tremor score	0.018	0.731
Cranial tremor score	0.272	< 0.001^*^
Numbers of near falls or falls	0.246	< 0.001^*^
CMSE score	−0.301	< 0.001^*^
HARS score	0.163	0.002^*^
HDRS score	0.210	< 0.001^*^
Global PSQI score	0.211	< 0.001^*^

^*^Significant association.

**Figure 1:**
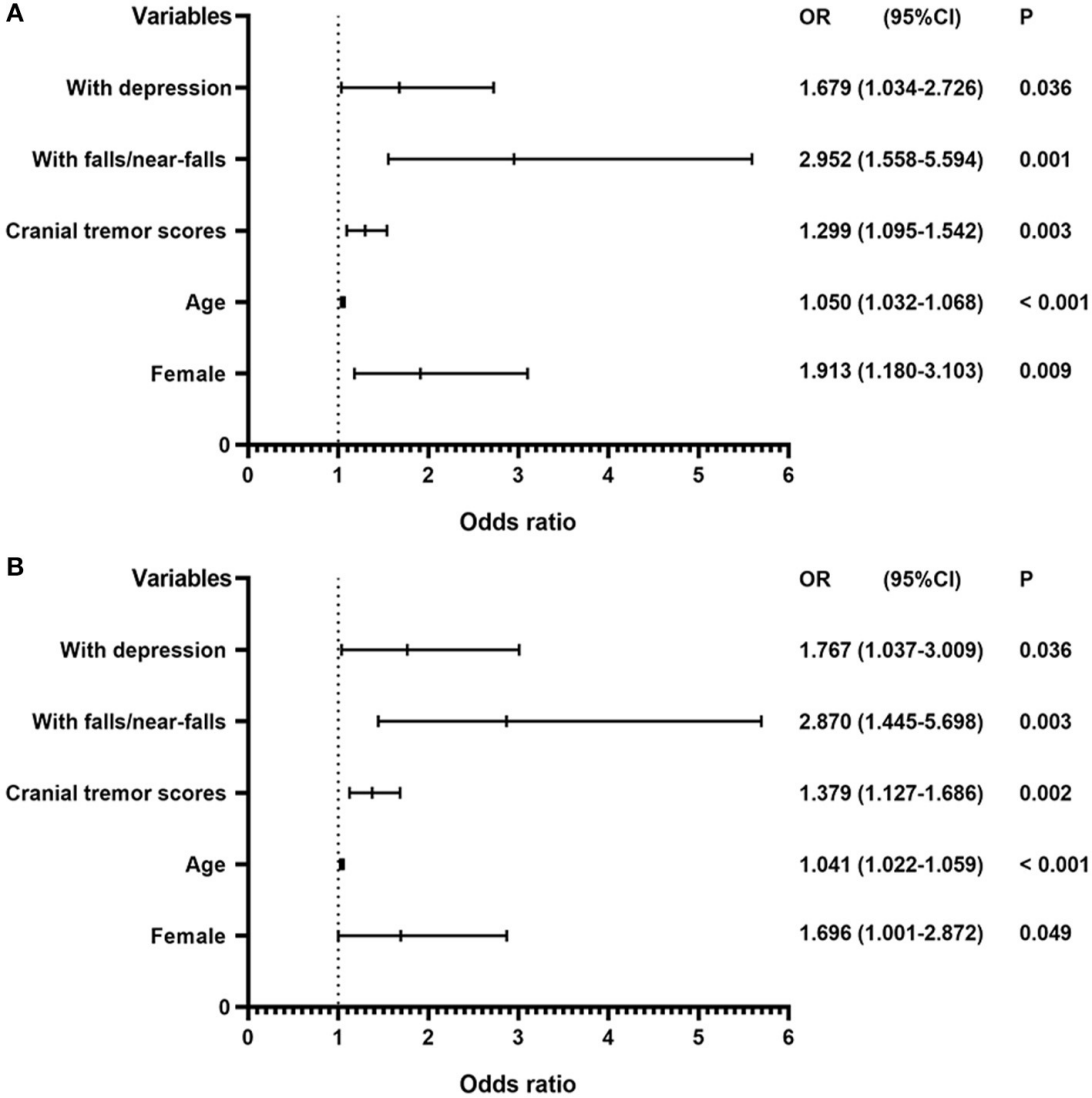
Risk factors associated with a-TG in patients with ET syndrome. **(A)** A model including all 358 patients with ET syndrome. **(B)** In a model (total ET syndrome: 292, a-TG: 128, n-TG: 164), we removed patients who had mild osteoarthrosis, or who were currently taking medications that may directly or indirectly affect balance, gait, or increase the risk of falls (medicines are shown in Supplementary Table 1). ET, essential tremor; CI, confidence interval; OR, odds ratio. Odds ratios and 95% confidence intervals are presented to show the risk factors associated with a-TG in patients with ET syndrome.

In regression models adjusted for confounding variables, we identified that female sex (OR = 1.913, 95% CI: 1.180–3.103), age (OR = 1.050, 95% CI: 1.032–1.068), cranial tremor scores (OR = 1.299, 95% CI: 1.095–1.542), a history of falls or near-falls within the past year (OR = 2.952, 95% CI: 1.558–5.594), and the presence of depressive symptoms (OR = 1.679, 95% CI: 1.034–2.726) were associated with the occurrence of a-TG in patients with ET syndrome. Similar results were found after we removed 66 patients who presented with osteoarthrosis and/or were currently taking medicines that may have affected balance and gait (Figure 1).

## 4. Discussion

Through the evaluation of TG tests and related clinical characteristics of 358 patients with ET syndrome, we found that the incidence of a-TG in an ET syndrome population from Southwest China was 47.2%. We found that worse balance performance was associated with female, advanced age and the presence of cranial tremors. In addition, we revealed a novel finding that worse balance performance was significantly associated with the occurrence of falls or near-falls in patients with ET syndrome, and patients with a-TG had pronounced non-motor symptoms, among which depressive symptoms were independently related to a-TG.

Using a similar definition and evaluation method of a-TG compared to those employed in previous studies, the frequency of a-TG in our patients was within the range of incidences that have been reported in previous studies (30–70%) (2–5, 9). Except for the average age (60.9 years old) reported for the cohort by Lim et al. (18), the average age of patients in other studies is much older (68–77 years old) (2–5) than those of the patients reported in this study. Importantly, advanced age is an independent risk factor for poor TG performance (2–5, 18), which we also confirmed in our present study and which may explain the discrepancy in the incidence of a-TG.

For the first time, we explored the association between TG performance and the occurrence of falls/near-falls in patients with ET syndrome and found that the OR of an a-TG patient having a history of falls or near-falls was 2.952 (*P* = 0.001). In addition, we found that patients with a history of near-falls were more likely to experience falls, confirming that a near-fall can predict the occurrence of a fall and likely represents postural instability (24). Previous studies have shown that a decrease in balance confidence, gait speed of < 0.9 m/s, and cognitive decline are predictors of falls in patients with ET syndrome (25, 26), and our study reflected that the occurrence of falls in patients with ET syndrome may be associated with more severe and extensive lesions in balance-related pathways or structures, as TG tests can reflect abnormalities in the vermis, flocculonodular lobe, and/or cerebellar pathways linking to brainstem nuclei (15). In turn, TG abnormalities may be a predictor of fall risk in patients with ET syndrome.

Regarding non-motor symptoms, previous studies have found that ET syndrome patients with cognitive impairments have more gait and balance problems, such as more missteps in the TG test, as well as a greater number of falls (5, 25, 26). In our present study, patients with a-TG had lower CMSE scores. However, after adjustments for age and educational years, poor cognitive performance was not an independent risk factor for a-TG. This result may be because the included patients did not have dementia and were relatively young, and the CMSE scale is less sensitive to mild cognitive impairment (MCI).

Another interesting finding of the present study is that depressive symptoms were associated with a-TG in our patients. An association between depression and gait/balance has been reported in community-dwelling elderly individuals (12). A higher burden of depressive symptoms may increase the risk of fear of falling, falling, and poor performance in quantitative gait variables in elderly individuals (12, 27, 28). The relationship between depression and cognition may be an underlying link, as it has been reported that depression is a risk factor for dementia, and patients with depressive symptoms may manifest deficiencies in attention and execution, which may lead to difficulties in completing gait-related tasks (29–31). Furthermore, a-TG may be due to abnormalities in the vermis of the cerebellum. In addition to contributing to axial balance, the cerebellar vermis also communicates with limbic structures that are involved in emotional processing (15, 32). Moreover, one recent neuroimaging study reported that ET syndrome patients with depressive symptoms have structural changes in the amygdala, which innervates other brain regions that are involved in posture control (10, 11). In addition, depressed patients may lack self-confidence in physical activities, causing tension in the TG task. In turn, balance problems may lead to increased depressive symptoms.

The severity and frequency of anxiety, as well as poor sleep quality, were more prevalent in patients with a-TG in our present study, but these two symptoms were not independently associated with a-TG following multiple regression analysis. Anxiety and poor sleep share some common neural circuits with balance control, which may cause their concomitance with a-TG (11, 33). Neural pathways that process afferent visceral and vestibular information related to postural/balance control are also linked to the parabrachial nucleus network, which participates in generating anxiety and fear (11). Sleep disorders (especially sleep deprivation) lead to balance problems by affecting vestibular-related oculomotor circuits (33). Our patients with a-TG had longer sleep latencies and shorter sleep durations, which may lead to chronic sleep deprivation. In addition to having common anatomical structures associated with balance control, anxiety and sleep disorders also induce cognitive deficits, especially attention deficits, which induce a poor completion of the TG task (31). Moreover, a decline in balance may cause anxiety and fear in patients. Nonetheless, since cognitive impairment, depression, anxiety, and sleep disorders can co-occur in such patients, the effects of these non-motor symptoms on balance may interact with one another, or may be secondary to primary symptoms (e.g., depression). Indeed, there were correlations among the scores on these scales. Therefore, future studies in subgroups (such as patients with depression alone) may be needed to reduce the influence of the interaction of non-motor symptoms on the results. These results need to be confirmed by postmortem and/or neuroimaging studies.

Our present study found that a-TG was more likely to appear in patients with neck, voice, facial, and cranial tremors, and cranial tremor scores represented an independent correlated factor for a-TG. The association between cranial tremors and worse performance in the TG test has been reported previously (4, 5). In addition, another study also found that patients with head tremors have more near misses and lower scores on objective balance scales (34). Postmortem and neuroimaging studies have linked cranial tremors to more cerebellar lesions, especially within the vermis (35, 36). Therefore, the co-occurrence of balance/gait issues and cranial tremors may be related to the overlap of the neuroanatomy. In addition, the finding observed from the present study shows that female patients with ET syndrome have a higher susceptibility to balance impairment which may be due to the higher preponderance of cranial tremors in female patients.

The strength of the present study is that it included a relatively large sample size and employed detailed evaluations of clinical correlates with regard to balance (i.e., the TG test), motor symptoms, cognition, mental disorders, and sleep disturbances.

Our present study had several limitations. First, our single-center, cross-sectional design limited the deduction of causality, and our results may not be applicable to all populations. Furthermore, a-TG can also be present in other conditions, such as in patients with peripheral neuropathy or abnormal vestibular function. We only excluded such patients *via* their medical histories and physical examinations rather than using objective exams. Moreover, TG testing lacks a standardized, guideline-based protocol. In addition, falls or near-falls were retrospectively collected and may have consequently represented underestimates. Finally, the age of the a-TG group was higher than that of the n-TG group. Although we used multiple logistic regression to adjust for age on other factors, future studies should be carried out in patients of similar age groups to better exclude the influence of age on variables such as cognition.

## 5. Conclusion

We directly employed a-TG to detect the occurrence of a fall or near-fall in patients with ET syndrome and, for the first time, evaluated correlations among depression, anxiety, sleep disturbances, and gait abnormalities. We found that patients with a-TG were predominantly women, older, had cranial tremors, exhibited pervasive depressive symptoms, and were more likely to have a history of falls or near-falls. In summary, in ET syndrome patients with cranial tremors or depressive symptoms, we should devote additional attention to their balance ability to optimize disease management. Moreover, TG abnormalities may be regarded as a predictor of fall risk in patients with ET syndrome.

## Data availability statement

The raw data supporting the conclusions of this article will be made available by the authors, without undue reservation.

## Ethics statement

The studies involving human participants were reviewed and approved by Ethics Committee of West China Hospital of Sichuan University (2020-842). The patients/participants provided their written informed consent to participate in this study.

## Author contributions

HH: writing of the first draft of the manuscript. HH and YX: conception of the research project and statistical analysis. XY and YX: supervision. All authors: review, critique, organization, and execution.
