# Supplementary material for: Prevalence and risk factors for abnormal tandem gait in patients with essential tremor syndrome: A cross-sectional study in Southwest China
Source: Front Neurol. 2023 Feb 16;14:998205. doi: 10.3389/fneur.2023.998205 (PMC9978743; doi:10.3389/fneur.2023.998205)
Supplement: Supplementary file 1 [file Table_1.PDF]

Supplementary Material

Prevalence and risk factors for abnormal tandem gait in patients with essential tremor syndrome: a cross-sectional study in southwest China

Supplementary table 1. List of medications being used by patients recruited in this study that may affect balance, gait, or increase the risk of falls

Names
**Medications for hypertension**
Levamlodipine Besylate Tablets
Amlodipine Besylate Tablets
Nifedipine Controlled-release Tablets
Nifedipine Tablets
Felodipine Sustained Release Tablets
Losartan Potassium Tablets
Irbesartan Tablets
Irbesartan and Hydrochlorothiazide Tablets
Fosinopril Sodium Tablets
Perindopril Arginine Tablets
Metoprolol Tartrate Tablets
Bisoprolol Fumarate Tablets
**Medications for diabetes**
Glimepiride Tablets
Gliclazide Modified Release Tablets
Gliquidone Tablets
Metformin Hydrochloride Tablets
Acarbose Tablets
Isophane Protamine Human Insulin injection
**Medications for anxiety/depression/sleep disorders**
Escitalopram Oxalate Tablets
Venlafaxine Hydrochloride Sustained-Release Capsules
Fluoxetine Hydrochloride Capsules
Trazodone Hydrochloride Tablets
Mirtazapine Tablets
Clomipramine Hydrochloride Tablets
Amitriptyline Hydrochloride Tablets
Alprazolam Tablets
Clonazepam Tablets
